# Conventional and Basal Implantology: A Narrative Review of Current Evidence, Clinical Decision-Making, and Future Perspectives

**DOI:** 10.7759/cureus.113703

**Published:** 2026-07-31

**Authors:** Megha Joshi, Mayur Kaushik, Mohd Nazar Rana, Soumya Kaushik

**Affiliations:** 1 Periodontolgy, Subharti Dental College and Hospital, Swami Vivekanand Subharti University, Meerut, IND; 2 Periodontology and Implantology, Subharti Dental College and Hospital, Swami Vivekanand Subharti University, Meerut, IND; 3 Periodontology, Subharti Dental College and Hospital, Swami Vivekanand Subharti University, Meerut, IND; 4 Periodontology and Oral Implantology, Subharti Dental College and Hospital, Swami Vivekanand Subharti University, Meerut, IND

**Keywords:** bone remodeling, bone-to-implant contact, cortical bone, osseoadaptation, splinting implants

## Abstract

Dental implantology has transformed tooth replacement by offering reliable, functional, and aesthetic solutions. Conventional implants rely on adequate alveolar bone and often require staged procedures with delayed loading. They come in various designs with surface treatments to enhance osseointegration. Basal implants, in contrast, anchor into the dense cortical bone, enabling immediate loading without bone grafting. These single-piece implants have polished surfaces to reduce infection and require minimal surgical intervention. While basal implants offer faster treatment and are suitable for severe bone loss or compromised patients, they need expert handling and may be less aesthetic for single teeth. Both implant types have distinct roles in modern implant dentistry, addressing diverse clinical needs. The article provides a comprehensive overview of the evolving landscape of dental implantology, highlighting the contrasting principles and applications of cortical and cancellous implants.

## Introduction and background

Dental implantology has significantly transformed restorative dentistry by providing a reliable, functional, and aesthetically superior option for replacing missing teeth. These implants are alloplastic materials surgically inserted into or onto the jawbone, serving as stable anchors for prosthetic restorations. Since the evolution of modern implantology by Branemark [1] through the concept of osseointegration around dental implants in the 1950s, there have been remarkable advancements in implant materials, design, and surgical techniques, establishing them as the preferred solution for both partial and complete tooth loss. Today, dental implants not only restore chewing efficiency and help maintain jawbone integrity but also enhance facial appearance and overall patient satisfaction. Their long-term success is altered by various determinants including surgical skill, the quality of harbouring bone, implant type, and precise prosthetic planning. Recent advancements in dental implantology have led to the development of various implant systems tailored to meet diverse anatomical and clinical needs. Among these, conventional and basal implants represent two core approaches, each with distinct principles [1].

Conventional (Crestal) implants are placed in the alveolar bone and typically require sufficient bone volume for successful integration. In cases of bone loss, procedures such as grafting or sinus lifting are often necessary [2]. Conventional dental implants may be placed using either a two-stage (submerged) or one-stage (non-submerged) surgical protocol, depending on the clinical situation. In the two-stage approach, the implant is placed within the alveolar bone and covered by the mucosa during an initial healing period to allow undisturbed osseointegration. After sufficient healing, a second surgical procedure is performed to expose the implant and place a healing or prosthetic abutment before fabrication of the definitive restoration. This protocol is often preferred in cases with limited primary stability, compromised bone quality, or when bone augmentation has been performed, as it protects the implant during healing and supports predictable osseointegration. By contrast, the one-stage approach leaves a healing abutment exposed through the mucosa at the time of implant placement, eliminating the need for a second surgery. This approach may be selected when adequate primary stability and favorable soft tissue conditions are present. Prosthetic loading may be immediate, early, or delayed based on implant stability and the clinical scenario [2].

By contrast, basal implants engage the denser cortical bone, making them suitable for patients with severe bone resorption. Often designed as single-piece implants, they allow for immediate loading and eliminate the need for bone augmentation, offering a faster, more cost-effective solution-particularly beneficial for medically compromised patients or high-demand cases [3].

Consequently, considerable debate exists regarding the indications, predictability, long-term outcomes, and evidence base supporting each implant system. While both systems are effective, they differ in surgical technique, loading protocol, and biological anchorage. Conventional implants are well-established with long-term success, whereas basal implants are gaining acceptance for complex scenarios, especially in regions where quick and graft-free solutions are preferred [3]. With innovations in surface technology, digital planning, and guided surgery, dental implants now offer survival rates exceeding 95% over a decade. As technology progresses, implant dentistry continues to set new benchmarks in restoring function, aesthetics, and quality of life [2].

The aim of this review is to critically compare conventional and basal implant systems with respect to their biological principles, design characteristics, surgical protocols, clinical indications, advantages, limitations, and treatment outcomes, thereby providing clinicians with evidence-based guidance for implant selection.

## Review

Literature search strategy

A comprehensive literature search was conducted to identify relevant studies comparing conventional and basal dental implant systems. Electronic databases, including PubMed/MEDLINE, Scopus, Google Scholar, Web of Science, and Cochrane Library, were searched for articles published between 2000 and 2025. The search was performed using combinations of the following keywords and Medical Subject Headings (MeSH) terms: “dental implants”, “conventional implants”, “crestal implants”, “basal implants”, “cortical implants”, “osseointegration”, “immediate loading”, “implant survival”, “implant success”, and “atrophic jaw rehabilitation".

The inclusion criteria comprised systematic reviews, meta-analyses, randomized controlled trials, prospective and retrospective clinical studies, and relevant narrative reviews published in English that evaluated the biological, biomechanical, and clinical aspects of conventional and basal implant systems. Studies lacking sufficient clinical data, case reports with limited sample sizes, conference abstracts, unpublished manuscripts, and non-English publications were excluded. Relevant articles were screened based on title, abstract, and full-text evaluation to ensure their relevance to the objectives of this review.

Conventional implantology

**Figure 1:**
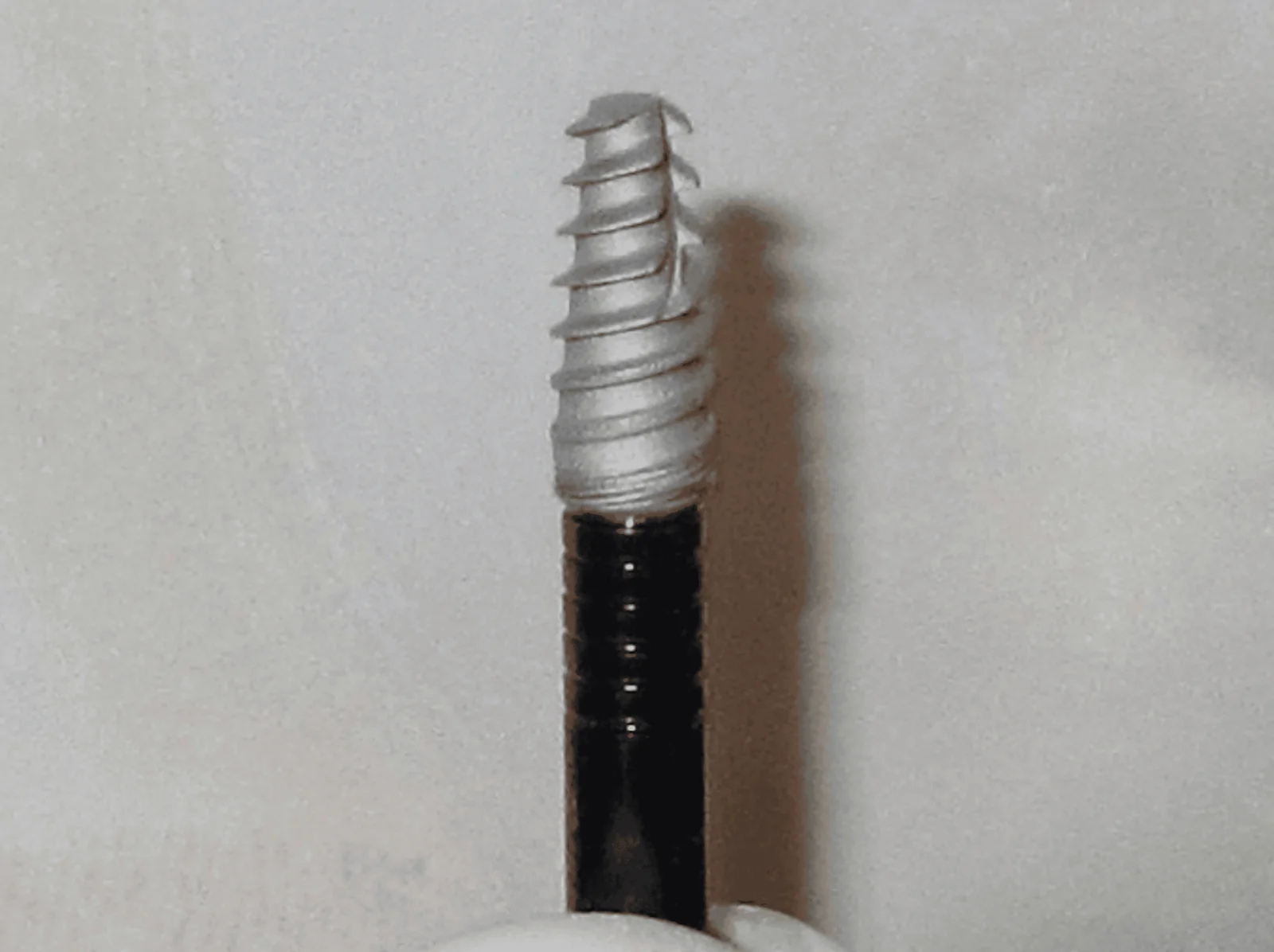
Conventional implant

Conventional, or crestal, implants are endosseous implants placed into the alveolar bone and are the most commonly used in current dental practice (Figure 1). They function as artificial tooth roots to support crowns, bridges, or dentures. Successful osseointegration, typically achieved over a three-to-six-month healing period, is critical and influenced by determining factors, such as quality of bone, surface and geometry of the implant, surgical technique, primary stability, and biocompatibility [2]. These implants require adequate bone volume and may necessitate bone grafting or sinus lift procedures in cases of deficiency. Supported by extensive clinical research, conventional implants offer a predictable and reliable solution for restoring both function and esthetics in suitable candidates. The macrodesign of conventional implants refers to the combination of macro-threads and micro-threads, which play a crucial role in achieving implant success by facilitating bone-to-implant contact (BIC). Dental implant systems are categorized based on their structural design, placement site within the jawbone, and loading protocol. Recognizing these classifications aids in choosing the most suitable implant type for specific clinical needs [2].

Evolution of conventional implants

Conventional dental implants, particularly endosseous implants, have evolved through multiple design modifications over time. Early implant designs included blade implants, introduced in the 1960s for use in narrow alveolar ridges. These implants, fabricated from titanium or chromium-nickel alloys, required high-speed drilling, which frequently resulted in excessive bone trauma, fibrous tissue formation, and compromised osseointegration, ultimately limiting their clinical acceptance. Pin implants, which are now largely obsolete, were designed to achieve stability through the insertion of multiple diverging pins. Although they offered the advantage of relatively easy removal with minimal bone loss, their clinical performance was hampered by complications such as bone necrosis and fibrous encapsulation. Disk implants [4], developed to provide resistance against vertical occlusal forces, similarly faced limitations related to surgical technique. The extensive bone preparation required for their placement often led to fibrous tissue interposition rather than true osseointegration, thereby affecting long-term success [4]. In an effort to enhance osseointegration, root-form (cylindrical) implants were introduced, utilizing press-fit designs and surface modifications. Despite these advancements, early cylindrical implants demonstrated comparatively lower survival rates and higher complication profiles [4].

Currently, screw-shaped implants represent the most widely used implant design due to their superior primary stability, predictable osseointegration, and versatility. These implants are available in one-piece and two-piece configurations, narrow-diameter options for reduced ridge width, and bone-level or tissue-level designs to suit diverse clinical scenarios. Furthermore, tapered implant designs, which mimic the natural root morphology of teeth, provide improved adaptation to the osteotomy site, enhanced primary stability-particularly in softer bone-and more favorable force distribution, while minimizing the risk of cortical bone perforation [5].

Albrektsson et al. [6] recognized the implant surface as one of six critical factors influencing successful osseointegration. Since this discovery, there has been growing emphasis on modifying surface topography and chemistry to enhance bone healing and shorten the time between implant placement and functional loading. As a result, surface engineering has become a highly active area of research within implant dentistry. However, the vast array of current manufacturing techniques has made it increasingly challenging to categorize implant surfaces based solely on their modification methods.

The surface microstructure of dental implants, mainly the roughness, plays a crucial role in promoting osseointegration. It facilitates cell adhesion, fibrin retention, and osteogenic cell migration. Surface roughness is often measured by Ra (2D) and Sa (3D) [5], with an Ra of 1-2 µm considered optimal for bone integration. Implant surface modifications are categorized into additive and subtractive techniques to enhance osseointegration [7]. Additive methods involve depositing materials like hydroxyapatite (HA), titanium plasma spray (TPS), and calcium phosphate coatings to promote bone bonding, although issues like degradation and bacterial retention may occur. Anodization thickens the oxide layer, improving surface energy and cellular response. By contrast, subtractive techniques such as acid etching, sandblasting, and laser treatment remove surface layers to create micro-roughness. These methods boost osteoblast activity, enhance bone contact, and reduce contamination, making them crucial in optimizing implant success [7].

Components of a conventional implant system

The implant body (fixture) is the component that is surgically inserted into the alveolar bone and serves as the foundation for implant-supported restorations. It may be threaded or non-threaded, depending on the implant design and clinical requirements. The implant body consists of several important components. The crest module is the coronal portion that supports the prosthetic restoration, while the apex forms the apical end of the implant and is typically flat-ended with anti-rotation features such as vents or holes to enhance stability. The collar, often coated with hydroxyapatite (HA), promotes improved bone adaptation at the crestal region.

During the first-stage surgery, a cover screw is attached to temporarily seal the implant, preventing debris accumulation and soft tissue ingrowth during osseointegration. Following implant exposure in the second-stage surgery, a healing abutment (permucosal extension) is connected to extend through the soft tissue, facilitating gingival contouring and establishing a soft tissue seal around the implant. The definitive abutment connects the implant body to the prosthesis and functions similarly to a prepared natural tooth. Abutments may be classified as screw-retained, cement-retained, or attachment-retained, depending on the method of prosthesis retention, and are available in straight or angled designs to accommodate varying implant angulations and optimize prosthetic alignment and esthetics. During the impression procedure, a transfer coping (impression coping) accurately transfers the three-dimensional position of the implant to the working cast. A laboratory analog (implant analog), which is a replica of the implant body, is then incorporated into the cast to facilitate prosthesis fabrication. Finally, a prosthetic coping serves as a thin, precisely fitting connector between the abutment and the definitive prosthesis, particularly in screw-retained restorations, ensuring accurate fit and stability [8].

Surgical protocols and healing following osseointegration

Surgical placement of two-piece implant systems may be performed using three different protocols: one-stage, two-stage, or immediate restoration/loading, depending on the clinical situation and treatment objectives. The procedure begins with a comprehensive preoperative evaluation, including detailed clinical examination and radiographic assessment using cone-beam computed tomography (CBCT) or orthopantomography (OPG) to evaluate crestal bone morphology and identify vital anatomical structures.

Following administration of local infiltration anaesthesia to achieve profound regional analgesia, a sub-crestal mucoperiosteal incision is made, and a full-thickness mucoperiosteal flap is elevated to provide adequate access to the alveolar bone. Osteotomy preparation is performed sequentially, beginning with a pilot drill and progressing through drills of increasing diameter under copious sterile saline irrigation to prevent thermal osteonecrosis. The endosseous implant fixture is then inserted manually or using a low-speed, high-torque motor and positioned at or slightly below the crestal bone level. In the one-stage protocol, a transmucosal healing abutment is placed immediately, whereas in the two-stage protocol, a cover screw is secured, and the soft tissues are repositioned to allow submerged healing before a second-stage surgical exposure [9].

When immediate loading is indicated, the implant and abutment are placed simultaneously and restored with a provisional prosthesis. Wound closure is achieved with resorbable or non-resorbable sutures, with non-resorbable sutures typically removed after seven to 10 days. Postoperative management includes the prescription of systemic antibiotics, analgesics, and chlorhexidine gluconate mouth rinses, along with regular follow-up visits to monitor osseointegration and peri-implant soft tissue healing and a healing phase, i.e, an osseointegration period of approximately three to six months before definitive prosthetic rehabilitation [9,10].

Bergludh et al. [11] described the model for the different phases of wound healing that are involved in the process resulting in osseointegration. They observed that the initially empty wound chamber became occupied with a coagulum and granulation tissue that was replaced by a provisional matrix. The newly developed bone along the lateral edge of the prepared bony site appeared to integrate seamlessly with the existing native bone. However, areas of woven bone were also observed on surfaces located further from the original bone. Over time, this initial bone was gradually replaced by more mature parallel-fibered and/or lamellar bone along with marrow tissue. Within one to two weeks, the bone that initially provided the implant’s mechanical stability began to undergo resorption and was subsequently substituted by newly formed, viable bone [11].

The osseointegration process following placement of an implant occurs in distinct phases. The initial healing phase begins immediately after insertion and resembles the biological process of fracture healing. This phase is marked by inflammation, bone resorption, and the release of growth factors, which recruit osteoprogenitor cells to the site [8]. Osteoblasts then begin forming new bone directly on the implant surface. During this time, initial stability is provided by the torquing of the implant with the surrounding alveolar bone, determined by implant design and surgical precision. As healing progresses, secondary stability develops through osseointegration, where new bone forms around the implant, anchoring it biologically. The subsequent bone remodeling phase includes a “stability dip,” where primary stability declines before sufficient secondary stability is established. Advanced surface modifications and loading protocols aim to reduce this dip. A key indicator of successful integration is BIC, with an ideal range of 60-70% indicating favorable outcomes [8]. Resonance frequency analysis (RFA) serves as a valuable clinical tool for assessing the condition of the implant-bone interface at various stages of treatment and during follow-up. It effectively evaluates implant stability by measuring the stiffness of the interface between the implant and surrounding bone. RFA readings can be affected by several factors, including the duration of the healing period, the density of the bone, and the extent of the implant’s exposure above the alveolar crest [12].

Albrektsson [13] in 1986 established key benchmarks for evaluating dental implant outcomes. These include the absence of implant mobility, no evidence of radiolucency on radiographs indicating peri-implant bone loss, and crestal bone loss limited to a minimum of 0.2 mm annually after the first year of function. In addition, the dental implant site should be free of persistent pain, infection, neuropathies, or paraesthesia. Based on these parameters, a successful implant should achieve a survival rate of at least 85% over five years and 80% over a ten-year period, reflecting long-term clinical viability and patient satisfaction [13].

Advantages and limitations of conventional implants

Conventional dental implants are widely recognized for their clinical reliability and predictability, offering a long-term success rate exceeding 90-95% over a 10-year period. Their ability to achieve predictable osseointegration ensures a robust bone-to-implant interface, which contributes significantly to the functional stability and longevity of the prosthesis. Moreover, they promote alveolar bone preservation and remodeling, thereby enhancing both functional and aesthetic outcomes. These implants are remarkably versatile, making them apt for a variety of clinical indications, comprising the replacement of single teeth, multiple teeth, or full arches. They are available in various diameters, lengths, and surface modifications, allowing for customization based on individual anatomical and clinical requirements [8].

Despite their advantages, conventional implants do present certain clinical limitations. Successful placement is highly dependent on the presence of sufficient alveolar bone volume, which may necessitate additional grafting procedures in cases of resorbed ridges. The healing period is typically protracted, often requiring 3 to 6 months before the final prosthetic phase can be initiated. Furthermore, the cost of treatment may be substantial due to the involvement of multiple surgical and restorative stages. There is also a potential for biological and mechanical complications, such as peri-implant infection, screw loosening, and, in rare cases, implant failure [8].

Basal (cortical) implantology

**Figure 2:**
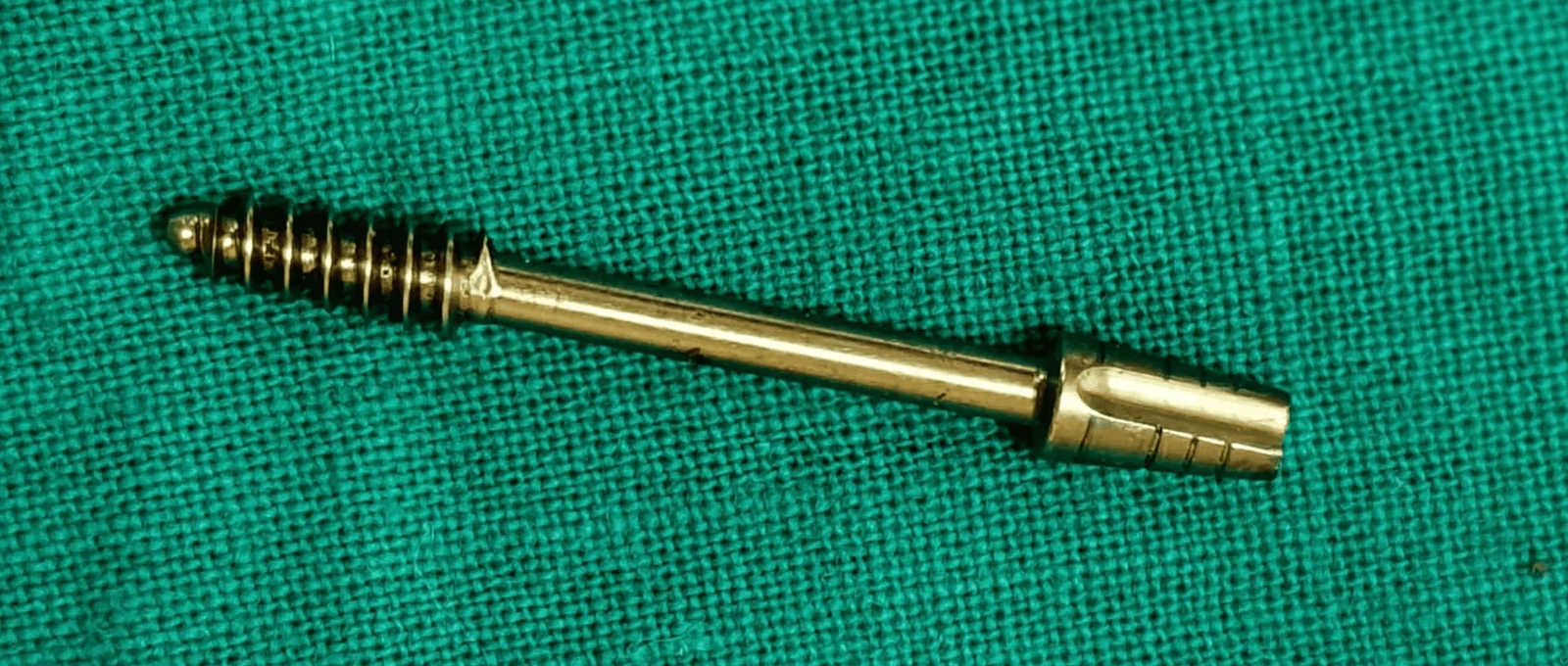
Basal implant

Basal implantology (also known as bicortical or cortical implantology) (Figure 2) is a dental implant system that utilizes the dense basal cortical bone of the jaw for implant retention, unlike conventional implants that rely on the softer alveolar bone. It is often referred to as “orthopedic implants” due to the principles adopted from orthopedic surgery [14]. It offers stronger load-bearing capacity due to cortical bone’s density. The rationale behind basal implantology lies in the natural process of bone resorption following tooth loss, where the alveolar bone, the primary support for teeth, gradually diminishes. By contrast, the underlying basal bone remains more stable and has an enhanced anti-resorption tendency due to its rigid, cortical framework. Basal dental implants are designed to anchor into this robust basal bone, providing a durable and infection-resistant solution. Their engagement with highly mineralized bone ensures excellent mechanical stability, making them a reliable long-term option, especially in cases with severe bone loss or compromised alveolar support [14].

Evolution of basal implants

The evolution of basal implantology can be traced back to 1972, when Jullietin JM [14] introduced the first single-piece implant, thereby establishing the conceptual foundation for modern basal implant systems. A significant milestone was achieved in 1997, with the development of lateral basal implants, commonly known as disk implants, by Ihde [15], which were specifically designed to achieve enhanced stability in patients with severely resorbed or compromised alveolar bone. Subsequent advancements in 2002 led to the introduction of a fracture-resistant base plate design, incorporating strategically engineered bending zones within the implant shaft. This innovation improved implant flexibility and facilitated more favorable biomechanical stress distribution under functional loading conditions [15]. In 2003, the advent of fully polished implant surfaces represented a pivotal development in basal implantology. This surface modification significantly reduced peri-implant inflammatory responses and enhanced soft tissue integration, particularly in sites affected by infection or compromised bone quality. Building upon these foundational developments, the period following 2005 witnessed the transformation of lateral basal implant concepts into screw-type basal implant systems. This evolution led to the emergence of advanced designs, such as the basal cortical screw (BCS) and basal osseointegrated screw (BOS) implants. Collectively, these innovations marked a paradigm shift in implant dentistry, enabling predictable immediate loading protocols and substantially expanding treatment options for patients with limited or deficient alveolar bone [15].

Indications and contraindications of basal implants

Basal implants are indicated in patients with multiple missing teeth or those requiring multiple extractions, in cases of failure of conventional two-stage implant systems, and when bone augmentation procedures have failed or are contraindicated. They are particularly beneficial in patients with severe alveolar bone atrophy characterized by extremely thin alveolar ridges or inadequate bone height and/or width, where bone grafting is either not feasible or contraindicated. Basal implants are also indicated when immediate loading protocols are desired in compromised bone conditions. However, they are contraindicated in patients who are unable to achieve balanced mastication due to neuromuscular disorders or muscular dysfunctions affecting occlusion, as well as in individuals with recent or unstable systemic conditions such as myocardial infarction or cerebrovascular accident (stroke) [15].

Classification of basal implants (based on morphology)

Based on their morphology, basal implants are classified into disk-form implants, including the transosseous implant (TOI), basal osseointegrated implant (BOI), and lateral implant; screw-form implants, comprising the bi-cortical screw, compression screw (KOS), and the combined KOS Plus implant; plate-form implants, including the BOI-BAC and BOI-BAC2 designs; and other specialized forms, such as the tuberopterygoid (TPG) implant and the zygoma screw implant (ZSI) [16].

Parts of a basal implant

Single-piece basal implants are characterized by a monoblock design in which the implant body and abutment are integrated into a single unit, thereby eliminating the implant-abutment interface and reducing complications associated with microgaps, screw loosening, and component failure seen in multi-piece implant systems. The implant surface is highly polished to minimize bacterial plaque accumulation and reduce the risk of peri-implant inflammation. The implant body is slender with wide thread turns, which enhance vascularity, facilitate optimal stress distribution, and increase bone-to-implant contact. Additionally, the implant neck is designed to permit abutment bending by approximately 15°-25°, depending on the implant length, when anchored in dense cortical bone, allowing prosthetic alignment without compromising implant stability. The polished neck further protects against bacterial adhesion, promoting long-term peri-implant tissue health [17].

Surgical technique and healing through osseoadaptation

Basal implant placement is typically performed using a minimally invasive surgical approach that varies according to the implant design. Compression screw, combination, and BCS implants require only a single osteotomy, which is prepared using manual drills to ensure precise and controlled bone preparation. Flap elevation is generally avoided to preserve the periosteal blood supply, and the implant geometry enables immediate loading without the need for extensive surgical exposure. By contrast, BOI require a more invasive technique involving lateral flap elevation and the preparation of a T-shaped osteotomy using disk drills. The implant is inserted laterally into the cortical bone, and the surgical site is subsequently closed with sutures. Owing to their engagement with dense cortical bone, basal implants achieve high primary stability, allowing immediate loading. However, as bone remodeling begins approximately 72 hours after implant placement, there is a temporary reduction in bone strength [18]. Therefore, rigid splinting of the implants is essential during this period to distribute occlusal forces evenly and prevent implant overload, thereby promoting predictable osseointegration and long-term clinical success [19].

Splinting is a critical component of the immediate loading protocol for basal implants, as it enhances biomechanical stability and ensures favorable load distribution. Implants are commonly splinted using rigid metal frameworks fabricated through intraoral welding (syncrystallization) to create a unified prosthetic structure [19]. BCS implants, which primarily depend on vertical cortical anchorage for stability, particularly require splinting to minimize functional stresses and prevent micromovement. Although BOIs exhibit greater resistance to vertical forces because of their lateral cortical engagement, they still require protection from lateral and shear forces through rigid splinting. Clinical evidence highlights the importance of this approach in achieving successful immediate loading outcomes. Lazarov [20] evaluated immediate functional loading of one-piece cortically anchored Strategic Implants® and reported a four-year cumulative survival rate of approximately 97.5%, based on the clinical performance of 1,019 BCS® implants, demonstrating the predictability and long-term success of splinted basal implant systems [21].

Advantages and limitations of basal implants

Basal implants offer several clinical advantages owing to their engagement with the dense, highly mineralized, and infection-resistant basal cortical bone, which provides excellent primary stability and facilitates the transmission of occlusal forces. Their polished implant surface and minimal mucosal penetration reduce bacterial adhesion and lower the risk of peri-implant infections. An important advantage is the elimination of bone grafting procedures, significantly shortening the overall treatment duration while enabling immediate functional loading, thereby avoiding any edentulous phase. Basal implants are well suited for one-stage surgical protocols and permit simultaneous tooth extraction and implant placement, even in infected extraction sites, making them particularly beneficial in complex or compromised clinical situations. Furthermore, because treatment is completed rapidly, they require comparatively less patient compliance [22].

Despite these benefits, basal implants have certain limitations. They may produce compromised esthetic outcomes in single-tooth anterior restorations or in cases requiring an ideal gingival contour and emergence profile. Their placement is highly technique-sensitive and demands advanced surgical expertise and comprehensive knowledge of maxillofacial anatomy. In patients with adequate alveolar bone volume, their use may result in unnecessary removal of healthy bone. In addition, inadequate biomechanical planning or improper occlusal force distribution may lead to overload osteolysis, potentially compromising long-term implant stability and clinical success [22].

Discussion

Dental implantology has evolved to address diverse clinical scenarios, leading to the development of both conventional and basal implant systems. Conventional implants, typically two-piece designs, rely on osseointegration within the alveolar bone and often necessitate sufficient bone volume, sometimes requiring bone augmentation procedures [13]. These implants have demonstrated high success rates, especially in patients with adequate quality and quantity of bone. Basal dental implants, on the other hand, are single-piece systems anchored in the dense cortical bone, allowing for immediate loading and eliminating the need for bone grafting [16].

There exist some mechanical and clinical differences between the conventional and basal implants (Table 1). While basal implants offer advantages in terms of immediate loading and suitability for compromised bone conditions, they require a high level of surgical expertise and detailed anatomical knowledge [23]. The aesthetic outcomes for single-tooth replacements may be less favourable compared to conventional implants. Furthermore, improper force distribution in basal implants can lead to overload osteolysis [24].

**Table 1:** Comparison between conventional and basal implants

	Conventional implant	Basal implant
Indications	Used for single or multiple unit restoration in adequate bone tissue.	Used for multiple unit restoration especially in extraction socket allow placement in bone deficient in height and width.
Mechanism	Osseointegration: “The formation of a direct interface between an implant and bone, without intervening soft tissue"	Osseo-adaptation: Cortical anchorage of thin screw implants (bicortical screws, BCS). Excellent primary stability can be obtained along the vertical surfaces of these implants with no need for corticalization. Only “osseoadaptation” occurs.
Loading protocol	Delayed loading: three to six months	Immediate loading: 72 hours
Implant procedure	Very often more complex surgical procedures are necessary, spread over two to three sittings in a period of three to six months (implant placement, healing screw placement, and abutment placement)	Single sitting surgical procedure and very often flapless (no open surgical procedures are necessary).
Armamentarium	Complex – a wide array of instruments are required for placement of two-piece implants	Simple – the implant surgery kit is very simple with very few instruments.
Cost	Expensive	Cost effective
Basic design	Two- piece system	One-piece system
Bone	Crestal alveolar bone, bone is more prone to resorption	Basal bone more dense, mineralized and less prone to bone resorption
Flexibility	Rigid	Bendable
Placement in the jaws	Good quality bone	Atrophied jaw
Fit with gums	Fits well after healing	May have gaps over time

Conversely, conventional implants, with their established protocols and extensive supporting evidence, remain the standard of care in implant dentistry. They are particularly advantageous in cases where esthetic outcomes are paramount, and sufficient bone volume is available [14]. Basal implants effectively restore masticatory function, esthetics, and speech, while also enhancing a patient’s self-confidence and overall quality of life [25]. However, replacing a failed basal implant can be challenging. Patients may experience more intraoperative discomfort and longer surgical duration compared to conventional methods, and there may be minor bone loss and recession of the surrounding gum tissue [26]. While basal dental implants have shown promising results in cases with deficient bone [27], there is insufficient evidence to recommend them over conventional (crestal) implants and hence requires further research in terms of their clinical efficacy.

The future of implant dentistry is expected to be driven by advancements in digital technologies, biomaterials, and patient-specific treatment planning. The integration of artificial intelligence (AI), three-dimensional imaging, and computer-guided implant surgery may improve diagnostic accuracy, surgical precision, and prosthetic outcomes. For conventional implantology, ongoing developments in implant surface modifications, biomimetic materials, and minimally invasive surgical protocols may further enhance osseointegration and reduce healing periods. In basal implantology, future research should focus on well-designed prospective clinical trials and long-term comparative studies to establish stronger evidence regarding survival rates, biomechanical behavior, and patient-centered outcomes compared with conventional implant systems.

In addition, standardized reporting protocols are needed to improve the quality and comparability of evidence related to basal implants. Advances in digital workflows, immediate loading protocols, and customized implant designs may expand treatment options for patients with severe alveolar atrophy while maintaining predictable outcomes. Ultimately, the future direction of implant therapy is likely to emphasize individualized treatment planning, where implant selection is based on anatomical conditions, systemic health, functional demands, and evidence-based clinical decision-making rather than a single preferred implant philosophy.

## Conclusions

Dental implants bridge the gap between function and aesthetics, making them an indispensable solution in modern dentistry. Innovations in implant surfaces, immediate loading, and digital planning have improved success rates and patient outcomes. Conventional implants depend on adequate alveolar bone and frequently need bone grafting but have proven to be successful over time. Meanwhile, basal implants secure themselves in the dense cortical bone, enabling immediate loading without the need for augmentation, making them suitable for patients with significant bone loss. Selecting the appropriate implant depends on individual patient factors such as bone quality and health status. Successful outcomes require collaboration between patient compliance and professional care, including regular maintenance. Overall, implants restore function, appearance, and quality of life, representing a major advancement in dental rehabilitation.

## Figures and Tables

**Figure 3:**
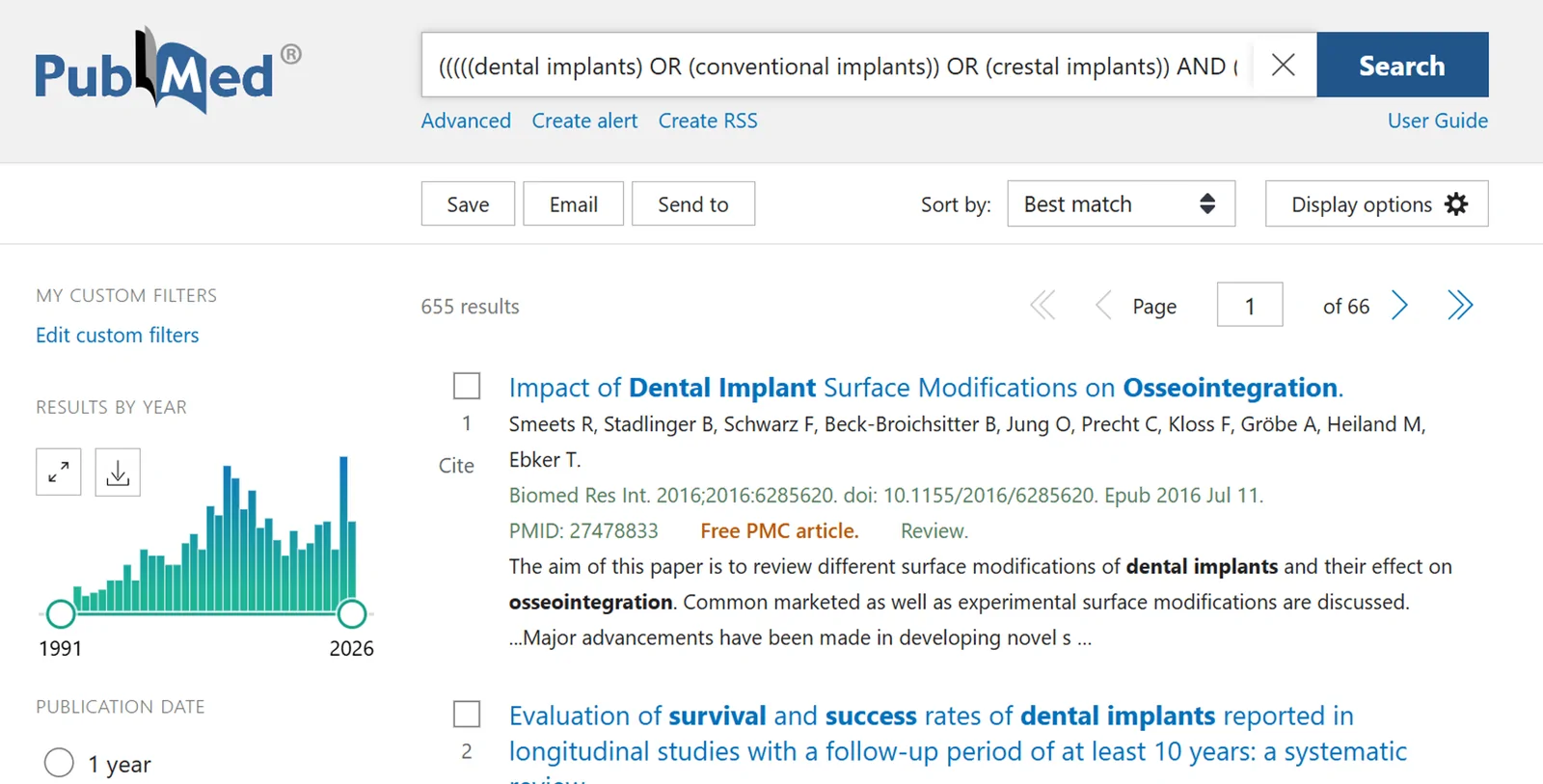
screenshot of search result

**Figure 4:**
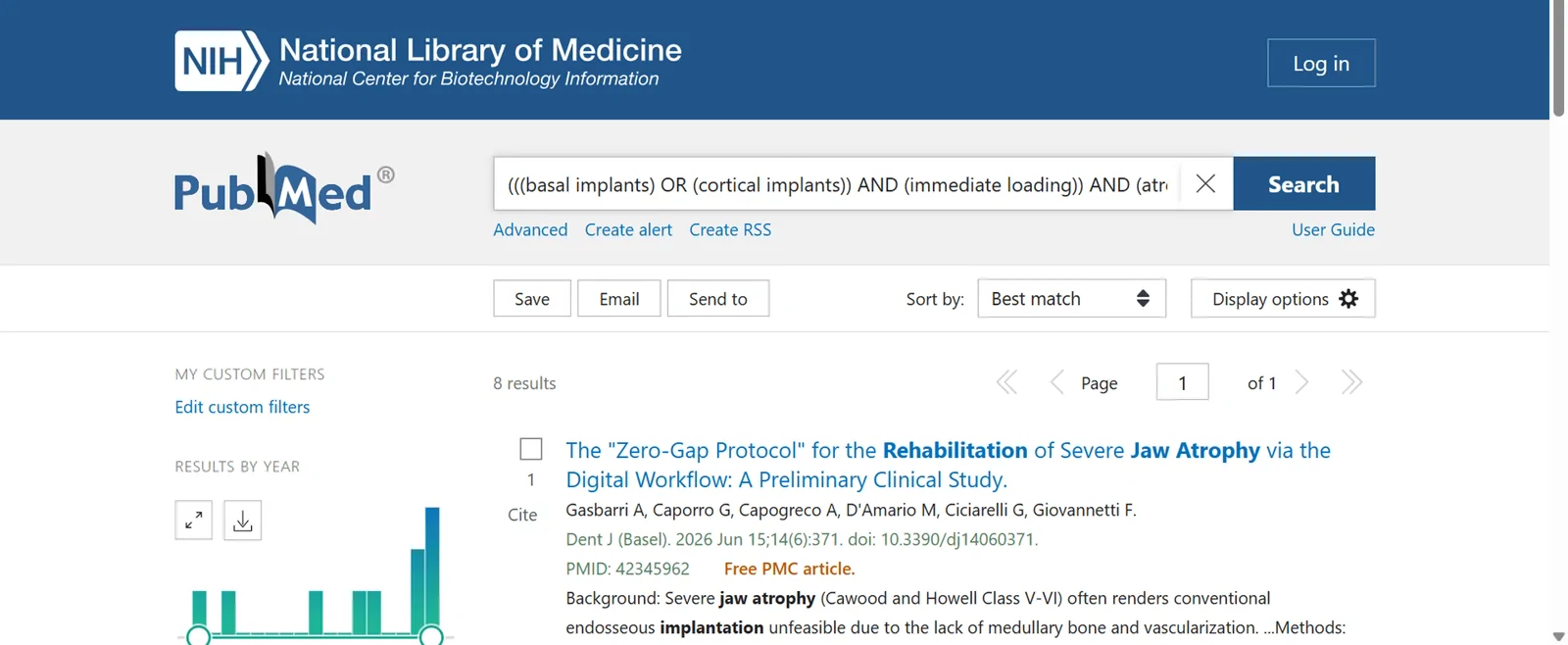
screenshot of search result
